# Esculetin Inhibits Fat Accumulation Through Insulin/Insulin-like Growth Factor- and AMP-Activated Protein Kinase-Dependent Pathways in *Caenorhabditis elegans*

**DOI:** 10.3390/nu17091565

**Published:** 2025-05-01

**Authors:** Aaron Taehwan Kim, Yeonhwa Park

**Affiliations:** Department of Food Science, University of Massachusetts, Amherst, MA 01003, USA; aaronkim@umass.edu

**Keywords:** esculetin, *Caenorhabditis elegans*, lipid metabolism, obesity

## Abstract

**Background:** Esculetin, 6,7-dihydroxycoumarin, is a bioactive compound found in various herbal plants, and is known to have health-beneficial properties including anti-obesity effects. However, there is a lack of in vivo studies to clearly determine esculetin’s role in lipid metabolism. **Objectives:** In this study, we studied esculetin’s effect on lipid accumulation using *Caenorhabditis elegans* and its underlying mechanisms. **Methods:** *C. elegans* were treated with esculetin (100 or 200 μM) for 48 h, and their triglyceride and protein levels were measured. Additionally, behavioral patterns such as pharyngeal pumping rate, body bending rate, body sizes, and locomotive activity were analyzed. Genetic dependencies were examined by utilizing mutant worms and testing relative gene expressions. **Results:** *C. elegans* treated with esculetin displayed significantly reduced fat accumulation compared to the controls without effects on the pharyngeal pumping rate, body bending rate, or locomotive activity. Esculetin’s fat-lowering effect was dependent on DAF-2 (insulin/insulin-like growth factor-1 [IGF-1] receptor homolog), DAF-16 (Forkhead box protein O homolog), and AAK-2 (5′-adenosine monophosphate-activated protein kinase [AMPK] catalytic subunit α2) in the mutant experiments. Esculetin also significantly increased the relative expression of downstream targets of DAF-16 (*hsp-16.2* and *sod-3*), AMPK-related genes (*aak-1* and *aak-2*), a sirtuin gene, *sir-2.1*, and a lipolysis-related gene, *atgl-1*. **Conclusions:** These findings suggest that esculetin inhibited fat accumulation in *C. elegans* and this effect was dependent on the insulin/IGF-1 and 5′-adenosine monophosphate-activated protein kinase signaling pathways.

## 1. Introduction

Esculetin, also known as aesculetin or 6,7-dihydroxycoumarin, is a natural bioactive coumarin derivative found in various herbal plants, such as *Viola mandshurica* W. Becker and *Fraxinus rhynchophylla* [1,2,3]. Esculetin is reported to have numerous health-beneficial properties, including anti-oxidant, anti-inflammatory, anti-cancer, anti-diabetic, and anti-apoptotic effects [4,5,6,7,8,9]. Additionally, esculetin is recognized as a naturally occurring inhibitor of lipoxygenases, such as rat platelet lipoxygenase and 5- and 12-lipoxygenases [10,11], and a previous study indicated that lipoxygenase inhibitors can reduce body fat accumulation in mice by suppressing lipoprotein lipase activity in adipocytes [12]. In fact, the anti-obesity effects of esculetin-rich *Viola mandshurica* W. Becker extracts were reported in mice [1,2,13]. Furthermore, reports have shown that esculetin reduced hepatic fat accumulation in both in vitro and in vivo studies [14,15,16,17,18,19]. Among these, Sim et al. [18] and Singuru et al. [19] reported that esculetin treatment significantly reduced adipose tissue mass in in vivo experiments. It has been consistently reported that esculetin reduces fat accumulation in 3T3-L1 adipocytes [20]. The mechanism behind esculetin’s effect on fat reduction depended on adenosine monophosphate-activated protein kinase (AMPK) [13,17,18,19,20,21]. Others have suggested that esculetin affects the insulin/insulin-like growth factor-1/phosphatidylinositide 3-kinase/protein kinase B pathway in human gastric cancer cells, promoting anti-proliferative and apoptotic activity [8]. Given the recognized role of the insulin/insulin-like growth factor-1 signaling (IIS) pathway in regulating energy metabolism, this could also represent a potential mechanism through which esculetin influences lipid metabolism [22,23,24]. Finally, we recently reported that esculetin reduced food preference behavior via the human μ-opioid receptor signaling pathway [25]. All these findings indicate that esculetin could be an effective anti-obesity compound. However, there is a lack of in vivo studies to clearly determine its role in lipid metabolism.

*Caenorhabditis elegans*, a transparent microscopic nematode, is a model organism used in life science research due to its simple structure and established genetic modifiability [26]. With over 65 percent of gene homologs to human genes related to disease, *C. elegans* serves as an excellent in vivo model for studying the anti-obesity effects of various phytochemical bioactive compounds, as well as the target genes and signaling pathways that are homologous to mammalian lipid metabolism pathways [26]. *C. elegans* accumulates lipid droplets primarily in the intestine, which is homologous to the mammalian liver and adipose tissue, and has previously been utilized to establish the effects of various phytochemicals and bioactive agents on lipid metabolism and to identify associated metabolic pathways [27,28,29,30,31]. Therefore, this study aimed to determine the anti-obesity effects of esculetin in *C. elegans* and the underlying mechanisms that affect lipid metabolism.

## 2. Materials and Methods

### 2.1. Materials

Esculetin (purity > 98%, CAS 301-01-1, PubChem CID 5281416) was purchased from Fisher Scientific (Waltham, MA, USA). Fluorodeoxyuridine (FUDR); carbenicillin, and ampicillin were purchased from Sigma-Aldrich Co. (St. Louis, MO, USA). Bleach was bought from a local store (Clorox Company, Oakland, CA, USA). Other chemicals were obtained from Fisher Scientific (Waltham, MA, USA) unless otherwise specified. *Caenorhabditis elegans* strains and *Escherichia coli* OP50 were purchased from *Caenorhabditis* Genetics Center (Minneapolis, MN, USA). The following mutant strains were purchased: Bristol strain N2 (wildtype), GR1307 *daf-16(mgDf50)*, CB1370 *daf-2(e1370) III*, VC199 *sir-2.1(ok434)*, GR2245 *skn-1(mg570)*, RB754 *aak-2(ok524)*, AGD397 *aak-1(tm1944) III;aak-2(ok524) X; uthEx202,* CE541 *sbp-1(ep79) III*, RB1716 *nhr-49(ok2165) I*, DG2179 *tub-1(nr2044) II*, BX107 *fat-5(tm420) V*, BX106 *fat-6(tm331) IV*, RB1668 *npr-19(ok2068)* *X. C. elegans* strains *npr-17(tm3210)*, and *aak-1(tm1944)* were obtained from National Bioresource Project for the Nematode (Tokyo, Japan). The following Taqman^®^ gene expression primers were purchased from Thermo Fisher Scientific Inc. (Middletown, VA, USA): *daf-2* (Ce02444340_m1), *daf-16* (Ce02422838_m1), *aak-1* (Ce02406989_g1), *aak-2* (Ce02404254_g1), *sir-2.1* (Ce02459017_g1), *let-363* (Ce02417512_m1), *hsp-16.2* (Ce02506738_s1), *sod-3* (Ce02404515_g1), *tub-1* (Ce02435687_g1), *kat-1* (Ce02434540_g1), *cebp-2* (Ce02421574_g1), *pod-2* (Ce02427721_g1), *fasn-1* (Ce02411650_g1), *fat-5* (Ce02488493_g1), *fat-6* (Ce02465318_g1), *fat-7* (Ce02477066_g1), *nhr-80* (Ce02421189_g1), *nhr-13* (Ce02471923_m1), *atgl-1* (Ce02406730_g1), *hosl-1* (Ce02494530_g1), *mdt-15* (Ce02406575_g1), *nhr-49* (Ce02412667_m1), *sbp-1* (Ce02453000_m1), *acs-11* (Ce02431951_m1), *ech-4* (Ce02438697_g1), *ech-1.1* (Ce02485968_g1), and a housekeeping gene *ama-1* (Ce02462726_m1).

### 2.2. Methods

#### 2.2.1. *Caenorhabditis elegans* Culture

*C. elegans* were grown on a nematode growth media plate at 20 °C in an incubator (DT2-MP-47, Tritech Research Inc., Los Angeles, CA, USA) as previously described, except for the *daf-2* mutant, which was grown at 15 °C [30]. The *C. elegans* population was synchronized using a bleaching solution and washed thrice with M9 buffer to collect eggs, followed by resuspending the eggs in S-medium buffer to achieve a density of ~1000 worms/mL [31]. The first-stage larvae (L1) were treated with ampicillin (100 μg/mL) and carbenicillin (50 μg/mL) and fed live *E. coli* OP50. At the fourth-stage larvae (L4), FUDR (0.12 mM) was added to the larvae to prevent the eggs from hatching. Adult *C. elegans* were treated with 0.1% dimethyl sulfoxide (DMSO) for the control or with esculetin (100 or 200 μM in 0.1% DMSO) for 48 h.

#### 2.2.2. Triglyceride and Protein Assays

After the two-day treatment, the *C. elegans* were washed with sterile Milli-Q water 5 times. Then, the *C. elegans* were resuspended in 0.05% Tween 20 and subjected to ultrasonication (Q125 Sonicator, Qsonica, Newtown, CT, USA) for triglyceride and protein assays [28,29]. The Infinity^TM^ Triglycerides Reagent (Fisher Diagnostics, Middletown, VA, USA) was used to measure triglyceride content, using glycerol as the standard. The Pierce^TM^ BCA protein assay kit (Thermo Fisher Scientific, Middletown, VA, USA) was used to measure protein content, with bovine serum albumin used as the standard. Absorbances were measured by a microplate reader (SpectraMax i3, Molecular Devices, Sunnyvale, CA, USA).

#### 2.2.3. Pumping Rate, Worm Body Size, and Locomotive Activities

The pharyngeal pumping rate, worm sizes (length and width), and speed were measured as previously described [30,32]. Low-peptone NGM plates with live *E. coli* OP50 lawns were prepared. Approximately 20 to 40 *C. elegans* were transferred to the plate and exposed to light for 10 min to allow the worms to adjust for observation. One-minute videos of the *C. elegans* on the observation lawns were recorded and analyzed using WormLab software (MSCOP-002, software ver. 3.1.0 64-bit, MBF Bioscience, Williston, VT, USA) to measure worm body size (length and width) and moving speed. Any *C. elegans* that paused during the one-minute recording were excluded from the data pool. The pumping rate of randomly selected *C. elegans* was measured for 30 s using an optical microscope. The bending rate was measured according to a previous report with minor modifications [33]. Briefly, the bending rates of 12 *C. elegans* were measured per test group, with one worm transferred at a time to a droplet of 10 μL S-medium buffer on a 30 mm Petri dish. After a 10 s adjustment period in the light at room temperature, the bending rate was measured for 30 s.

#### 2.2.4. Reverse Transcription–Quantitative Real-Time PCR (RT-qPCR)

RT-qPCR was performed as previously described [27]. Briefly, TRIzol^®^ reagent (Thermo Fisher Scientific Inc., Middletown, VA, USA) was used to extract RNA. cDNA was synthesized using a high-capacity cDNA reverse transcription kit (Thermo Fisher Scientific Inc.) with T100 Thermal Cycler (Bio-Rad Laboratories, Hercules, CA, USA). RT-qPCR was performed with a StepOnePlus^TM^ Real-Time PCR system (Applied Biosystems, Foster City, CA, USA). The comparative threshold cycle (Ct) was measured, and the gene expression was calculated with the 2^−ΔΔCt^ method.

#### 2.2.5. Statistical Analyses

Data are presented as mean ± standard error. A one-way analysis of variance, followed by Tukey’s post hoc test, was used to analyze the data (SAS version 9.4, SAS Institute Inc., Cary, NC, USA). Significance was determined at *p* < 0.05.

## 3. Results

### 3.1. Esculetin Decreased Fat Accumulation in Caenorhabditis elegans

**Figure 1:**
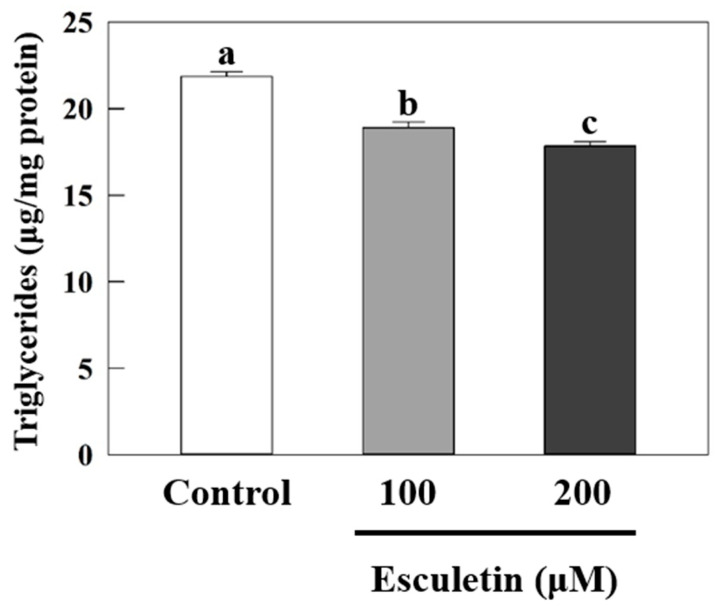
Esculetin reduced fat accumulation in wildtype *C. elegans*. Young adult wildtype worms were treated with esculetin (100 or 200 μM) for 2 days, followed by triglycerides and protein assays. Triglyceride content was normalized by protein content. Data are presented as means ± S.E. (*n* = 8). Means labeled with different letters (^a–c^) indicate significant differences at *p* < 0.05.

**Figure 2:**
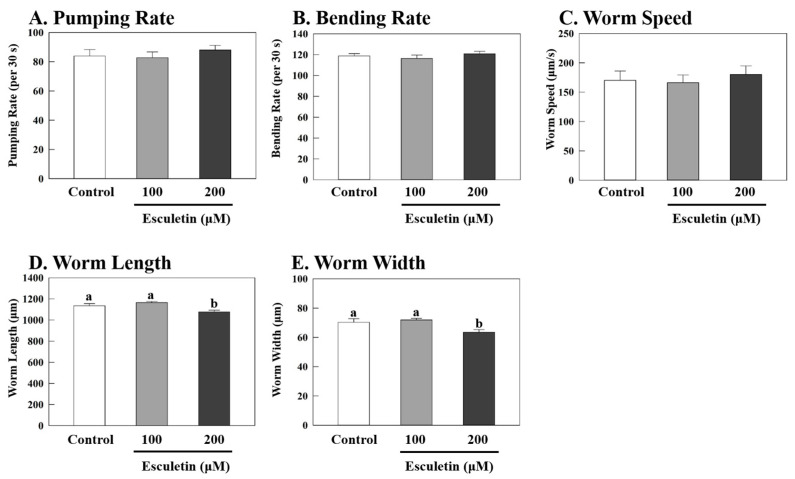
Feeding or locomotive behaviors of *C. elegans*. Adult wildtype worms were treated with 100 or 200 μM of esculetin for 2 days. (**A**) The pumping rate (n = 10) was measured using a microscope after transferring the worms to low-peptone NGM plates with live *E. coli* OP50. (**B**) The bending rate (n = 12) was measured after the worms were transferred to an S-medium buffer and measured with a microscope. (**C**–**E**) Worm speeds and sizes (*n* = 14–17) were measured by an automated tracking system after the worms were transferred to low-peptone NGM plates with live *E. coli* OP50. Data are presented as means ± S.E. Means labeled with different letters (^a,b^) indicate significant differences at *p* < 0.05.

Treatment with esculetin at 100 and 200 μM for 48 h reduced the triglyceride content in wildtype *C. elegans* by 14% (*p* < 0.001) and 18% (*p* < 0.001), respectively, compared to the control (Figure 1). To determine whether esculetin influenced food intake in *C. elegans*, we measured the pharyngeal pumping rate, which indicates the feeding behavior in this model (Figure 2A) [30]. Treatments with esculetin did not affect the pharyngeal pumping rate compared to the control, suggesting that the decrease in fat accumulation by esculetin was independent of food intake in this model (Figure 2A). Next, we measured the bending rate and worm speed, which reflect the worms’ activities and therefore represent energy expenditure (Figure 2B,C). Compared to the control, esculetin did not significantly alter either the bending rate or worm speed, indicating that any effect of esculetin on fat accumulation was independent of the worms’ energy expenditure (Figure 2B,C). Furthermore, we evaluated worm morphology and found that treatment with esculetin at 200 μM significantly reduced the worms’ width by 9% (*p* = 0.0336) and the worms’ length by 5% (*p* = 0.0381) compared to the control (Figure 2D,E). This reduction may be attributed to decreased fat accumulation, as previously observed in this model [27,30].

### 3.2. Esculetin’s Fat-Lowering Effect Was Dependent on Insulin/Insulin-like Growth Factor Signaling (IIS) Pathway

**Figure 3:**
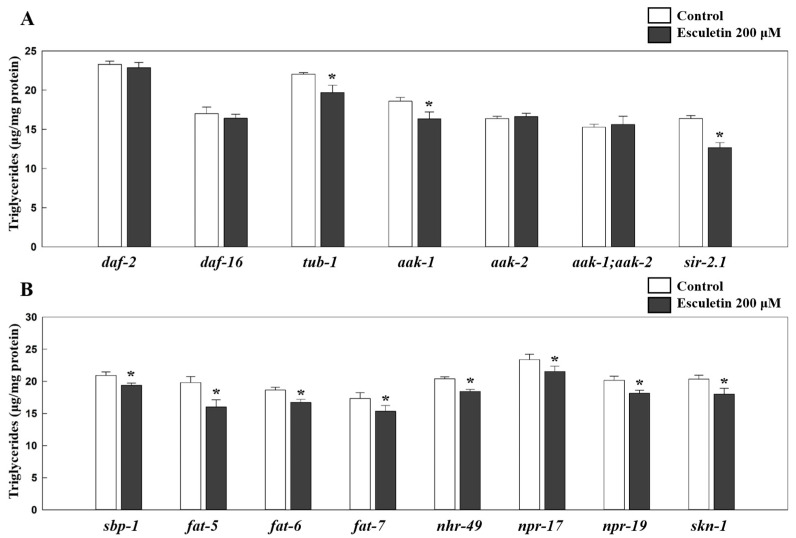
Effect of esculetin on fat accumulation in various *C. elegans* null mutants. Adult worms were treated with 200 μM esculetin for 2 days, followed by triglyceride and protein assays (**A**,**B**) (*n* = 4–8). The triglyceride levels were normalized to protein levels. Data are presented as means ± S.E. * indicates significant difference from the respective control at *p* < 0.05.

**Figure 4:**
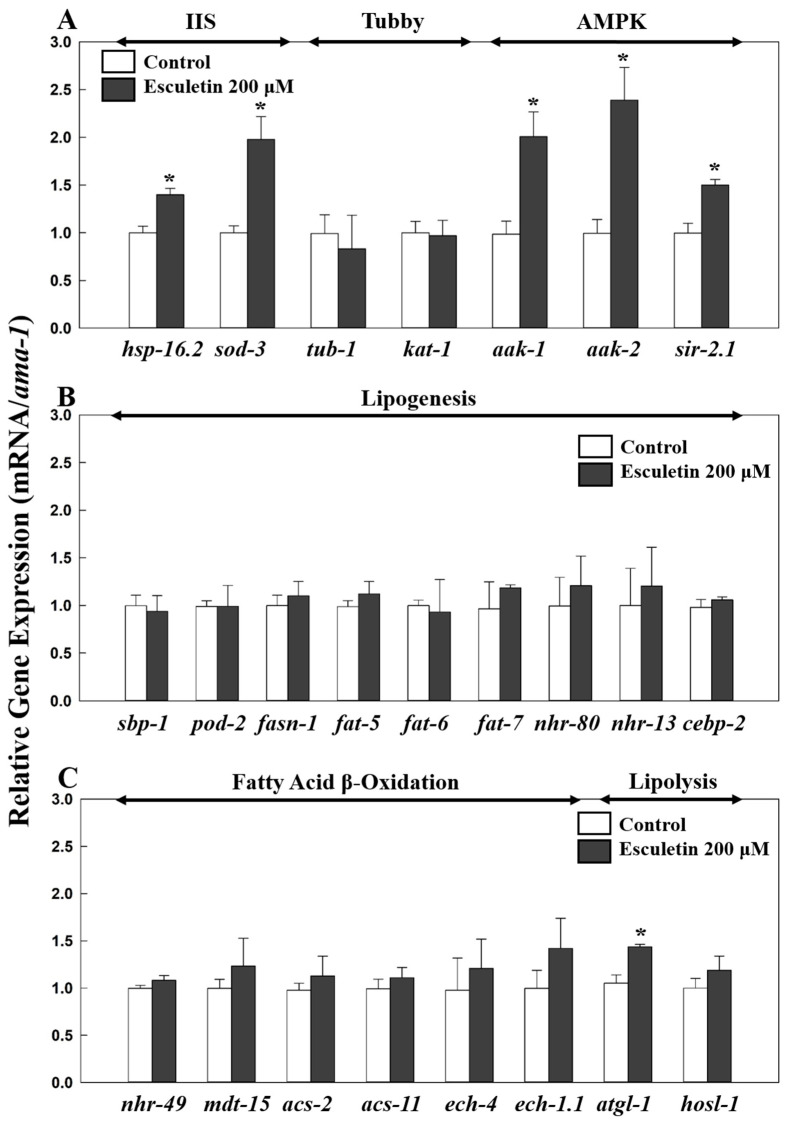
Effect of esculetin on fat accumulation-related gene expression on wildtype *C. elegans*. Adult wildtype worms were treated with 200 μM esculetin for 2 days, followed by RT-qPCR (**A**–**C**) (*n* = 3–8). Data are presented as means ± standard error. * indicates significant difference from the respective control at *p* < 0.05.

Next, to determine the target genes or pathways involved in esculetin’s effects on lipid metabolism, we tested esculetin in various known mutant strains associated with lipid metabolism (Figure 3) and gene expressions from the wildtype *C. elegans* (Figure 4). One of the major factors regulating fat accumulation in *C. elegans* is the IIS pathway [34]. While there are 40 insulin-like peptides in *C. elegans*, there is only one insulin-like receptor known as DAF-2 (abnormal dauer formation protein 2) [24]. When DAF-2 is activated, insulin receptor substrate proteins such as IST-1 (a homolog of insulin receptor substrate), AGE-1 (a homolog of phosphatidylinositol-3-OH kinase), PDK-1 (a homolog of phosphoinositide-dependent kinase), and AKT1/2 (a homolog of serine/threonine-protein kinase 1/2) are sequentially activated, ultimately leading to the phosphorylation of DAF-16 (a homolog of Forkhead box protein O), which prevents its entry into the nucleus, thereby inhibiting the cellular processes regulated by DAF-16 [35]. Esculetin’s fat-lowering effect in the wildtype *C. elegans* was abolished in the *daf-2-* and *daf-16*-null mutants, suggesting the involvement of *daf-2* and *daf-16* in esculetin’s effect on fat reduction (Figure 3A). Additionally, esculetin increased the relative expression of *daf-16* downstream genes, *hsp-16.2* (heat shock protein; 40%; *p* = 0.0153) and *sod-3* (superoxide dismutase; 51%; *p* = 0.0019) in the wildtype worms (Figure 4A). These results indicate that the fat-reducing effects of esculetin depend on the IIS pathway.

### 3.3. Esculetin’s Effect on Fat Reduction Was Independent of TUB-1

TUB-1, a homolog of the mammalian tubby protein, is known to play a role in fat storage through the neurological regulation of energy output [36]. Esculetin (200 μM) significantly reduced triglyceride levels compared to the control in the *tub-1*-null mutants (10%; *p* = 0.0489), indicating that esculetin’s effect on fat metabolism was independent of TUB-1 (Figure 3A). Similarly, esculetin also did not influence the expression of *tub-1* or its downstream target, *kat-1* (a homolog of mammalian acetyl-CoA acetyltransferase 1) in wildtype *C. elegans* (Figure 4A), suggesting that esculetin did not target the tubby signaling pathway to inhibit fat accumulation in *C. elegans*.

### 3.4. Esculetin’s Fat-Lowering Effect Was Dependent on AMP-Activated Protein Kinase (AMPK) Signaling

In *C. elegans*, there are two catalytic α subunits of adenosine monophosphate-activated protein kinase (AMPK), AAK-1 and AAK-2, which exhibit 52% and 40% similarity to human AMPKα1, and their kinase domains share 80% and 71% similarity with those of the human AMPKα1 subunit, respectively [37]. AMPK regulates energy homeostasis as a sensor for the ratio of adenosine monophosphate to adenosine triphosphate [37]. While AAK-1 and AAK-2 share sequence similarity, AAK-2 is regarded as the primarily functional AMPK in regulating lifespan and energy metabolism, whereas AAK-1 has a less prominent role [37]. Additionally, the AMPK signaling pathway extensively cross-interacts with the IIS pathway and is known to function alongside SIR-2.1 (the ortholog of human sirtuin 1) [38]. Esculetin at 200 μM significantly reduced the triglyceride content in the *aak-1*-null mutant (12%; *p* = 0.0401) and in *sir-2.1* (22%; *p* = 0.0023), but did not alter the fat content of the *aak-2*-null mutant and the *aak-1;aak-2* double mutant compared to the respective controls (Figure 3A). These results suggest that the effect of esculetin on fat reduction depended on *aak-2*. This is further supported by the increase in the expression level of *aak-2* (140%; *p* < 0.001) due to esculetin at 200 μM in the wildtype *C. elegans* (Figure 4A). However, esculetin also increased the expression of *aak-1* by 103% (*p* = 0.03740) and of *sir-2.1* by 50% (*p* = 0.0137) compared to the respective controls (Figure 4A).

### 3.5. Esculetin’s Effect Was Not Dependent on SREBP-1C nor PPARα Signaling

SBP-1 (a homolog of the mammalian sterol regulatory element binding protein-1C; SREBP-1C) is another key player in regulating fat metabolism and fatty acid synthesis [28,39]. SBP-1 regulates downstream targets such as *pod-2* (a homolog of acetyl-CoA carboxylase) and *fasn-1* (a homolog of fatty acid synthase) [38], which are key enzymes for lipogenesis. In the *sbp-1*-null mutants, treatment with esculetin significantly reduced the triglyceride content compared to the control (*p* = 0.0308) (Figure 3B). Furthermore, esculetin treatment did not change the relative expression of the *sbp-1* gene (Figure 4B). Additionally, esculetin did not affect the expression of SBP-1 downstream target genes, *pod-2* or *fasn-1*, in the wildtype (Figure 4B). Moreover, SBP-1 is known to regulate the expression of three downstream Δ9-desaturases, FAT-5, FAT-6, and FAT-7 (homologs of stearoyl-CoA desaturases) [40]. Treatment with esculetin decreased the triglyceride levels in the *fat-5-*, *fat-6-*, and *fat-7*-null mutants by 19% (*p* = 0.0384), 10% (*p* = 0.0272), and 12% (*p* = 0.0231), respectively, compared to the respective controls (Figure 3B), while the expressions of these genes remained unchanged in the wildtype *C. v* following esculetin treatment (Figure 4B). These results suggest that esculetin’s effect on lipid reduction in *C. elegans* did not involve the SBP-1/SREBP-1C signaling pathway.

*C. elegans* has a nuclear hormone receptor NHR-49 (the functional homolog of peroxisome proliferator-activated receptor alpha, PPARα), which is involved in fatty acid β-oxidation [30]. Esculetin at 200 μM significantly reduced the fat content in the *nhr-49*-null mutant (*p* = 0.0042) and did not affect the relative expression of *nhr-49* in wildtype (Figure 3B and Figure 4C). It is also known that NHR-49 collaborates with the nuclear receptors NHR-80 and NHR-13 to regulate fatty acid desaturase [41], all of which were unaffected by esculetin in the wildtype (Figure 4B). We further determined the expression of downstream targets of NHR-49: *mdt-15* (human mediator complex subunit 15), as well as *acs-2* (a homolog of human acyl-CoA synthetase 2), *acs-11* (an ortholog of human acyl-CoA synthetase 3), (an ortholog of human enoyl-CoA hydratase 2), and *ech-1.1* (a homolog of enoyl-CoA hydratase) (Figure 4C). Esculetin had no effect on the expression of *acs-2*, *acs-11*, *ech-4*, or *ech-1.1* (Figure 4C), suggesting that the reduction in fat accumulation due to esculetin was independent of the NHR-49 signaling pathways.

### 3.6. Esculetin’s Lipid-Reducing Effect Was Not Dependent on NPR-17, NPR-19, CEBP-2, or SKN-1

*C. elegans* contains functional homologs of opioid receptors (NPR-17) and cannabinoid 1 receptor (NPR-19), which play significant roles in food preferences and feeding behavior [42,43]. Activated opioid and cannabinoid signaling pathways have been reported to stimulate lipogenesis, and prior research has indicated that esculetin targets *npr-17* [25,44,45]. Esculetin significantly reduced triglyceride levels in *npr-17-* (*p* = 0.0257) and *npr-19*-null mutants (*p* = 0.0436) compared to their respective controls, suggesting that esculetin’s effect on fat accumulation was independent of the opioid or cannabinoid signaling pathways (Figure 3B).

The CCAAT-enhancer-binding proteins (C/EBP) are a family of transcription factors that play a crucial role in adipocyte development and lipid accumulation, and interact with various signaling pathways, including the SREBP-1C and AMPK pathways [46,47]. In *C. elegans*, which store most of their fat in the intestine, CEBP-2 (the homolog of mammalian C/EBP) regulates lipogenesis and body fat [38]. Esculetin did not alter the expression of *cebp-2*, indicating that its effect on fat reduction was independent of *cebp-2* (Figure 4B).

In *C. elegans*, SKN-1 is the mammalian nuclear factor erythroid 2-related 2 homolog, which is closely associated with the IIS and AMPK signaling pathways, and it plays a role in lipid metabolism and longevity [37,48,49]. Activated SKN-1 regulates various genes involved in energy metabolism and reduces fat storage [49,50]. Esculetin decreased fat accumulation in the *skn-1*-null mutant by 12% (*p* = 0.0218) compared to the control, suggesting that esculetin’s fat-lowering effect was independent of *skn-1* (Figure 3B).

### 3.7. Esculetin May Affect a Lipolysis-Related Gene

*C. elegans* contains lipases, *atgl-1* (a homolog of mammalian adipose triglyceride lipase; ATGL) and *hosl-1* (a homolog of the mammalian hormone-sensitive lipase E), both of which are targets of anti-obesity studies due to their roles in lipolysis [28,51]. Esculetin at 200 μM increased the expression of *atgl-1* by 36% (*p* = 0.0064) compared to the control, but it did not affect the expression of *hosl-1* (Figure 4C). These results suggest that esculetin may enhance lipolysis, contributing to its overall effects on fat reduction.

## 4. Discussion

Esculetin, also known as 4,6-hydroxycoumarin, is a coumarin derivative found in various traditional herbal plants, including *V. mandshurica* and *F. rhynchophylla* [1,2,3]. This study demonstrated that esculetin reduced fat accumulation in *C. elegans* without affecting energy expenditure or food intake. Furthermore, the current study revealed that the fat-reduction effect of esculetin involves the IIS and AMPK signaling pathways in *C. elegans*. These findings are consistent with previous reports indicating that esculetin reduces adipogenesis and fat accumulation [1,2,14,15,16,20]. This is the first study to report the in vivo effects of esculetin on reducing body fat accumulation using *C. elegans*.

This study identified that the fat-reducing effect of esculetin in *C. elegans* depends on the IIS pathway, specifically DAF-2, and its downstream target, DAF-16 [23,24]. It is also known that other kinases, such as AMPK can regulate DAF-16 [23,37]. The current results show that the effects of esculetin on fat reduction require AAK-2, although the activation of *aak-1* and *sir-2.1* may contribute to esculetin’s overall effects on lipid metabolism (Figure 3A). These observations are consistent with other reports indicating that esculetin affects the AMPK signaling pathway in adipocytes and hepatocytes [14,15,16,17,18,19,20]. In particular, Singuru et al. [19] reported that esculetin reduced adipose tissue via the AMPK-SIRT1 axis. Previous reports have suggested partial redundancy between *aak-1* and *aak-2*, which can partially compensate for each other’s absence regarding hypersensitivity to oxidative stress [52]. Another study has proposed the overlapping functions of these two isoforms; AAK-2 acts semi-dominantly in the germ line, while AAK-1 works additively with AAK-2 to suppress proliferation during the Dauer stage [53]. Therefore, it is not uncommon for a compound to target either AAK-1 or AAK-2 for its biological functions in this model. Overall, our results indicate that esculetin affects fat accumulation via IIS and AMPK, with contributions from AAK-1 and SIRT-1, consistent with previously reported mechanisms of the role of esculetin in lipid metabolism.

Previously, esculetin-rich *V. mandshurica* W. Becker water extract was found to reduce body fat in mice on a high-fat diet by inhibiting SREBP-1C [2,13]. Additionally, others have reported that esculetin suppressed the expression of lipogenesis genes by down-regulating SREBP-1C in hepatocytes, which plays a major role in cholesterol synthesis [16,18]. In *C. elegans*, which do not synthesize cholesterol, intestinal cells serve as the primary site for lipid metabolism, similar to mammalian hepatocytes and adipocytes [38,54]. However, in this study, the fat-reducing effects of esculetin in *C. elegans* were found to be independent of the SBP-1/SREBP-1C signaling pathway. Since AMPK can also regulate SREBP-1C, we speculate that the previous observation of esculetin’s effect on SREBP-1 may have been due to indirect effects on AMPK [47].

NHR-49, a functional homolog of mammalian PPARα, regulates fatty acid β-oxidation in *C. elegans* lipid metabolism [30]. In mammals, three PPARs (PPARα, PPARδ, and PPARγ) are structurally similar, sharing a conserved DNA-binding domain and ligand-binding domain [55]. Previously, Xia et al. [16] reported that esculetin activates AMPKα and its downstream target PPARα to improve lipid metabolism in palmitic acid/oleic acid-treated primary rat hepatocytes. The PPAR signaling pathway is also known to interact with the IIS pathway in lipid metabolism and insulin sensitivity in adipose tissue [56]. In this study, esculetin’s effect on inhibiting lipid accumulation in *C. elegans* depended on the AMPK and IIS pathways but did not involve the NHR-49/PPARα signaling pathway. Our findings indicate that the PPAR signaling pathway is not required for esculetin’s fat-lowering effect, which may be mediated through direct AMPK/IIS activation or alternative compensatory mechanisms.

The dose of esculetin used in the current study (100 and 200 μM) was determined based on a previous publication and the characteristics of the *C. elegans* model [25]. Previous in vitro and in vivo reports have utilized a wide range of esculetin doses: from 0.1 μM to 1 mM in various cells, and from 10 to 100 mg/kg in mice or rats [5,6,7,10,11,15,16,17,20,21,57,58]. Some studies have indicated that a 24 h treatment with esculetin at concentrations of 200, 400, 500, and 800 μM caused necrotic cell death in primary rat hepatocytes or 3T3-L1 mouse embryo fibroblasts [16,58]. Prolonged treatment (3 and 5 days) with 40 and 80 μM esculetin reduced colony formation in Lewis lung carcinoma cells [9]. In addition, one study reported that long-term supplementation of esculetin improved insulin resistance by activating AMPK signaling in obese mice [18]. Currently, there is no direct method to compare the doses used in *C. elegans* and cell cultures; however, some studies suggest that the *C. elegans* model requires higher concentrations of chemicals to achieve similar results compared to in vitro conditions [59] due to poor permeability or constant excretion [60,61].

The study’s limitations include the fact that the dose used in the current study cannot be directly translated to a functional dose for mammals. Even with the conserved similarities in physiology and biochemistry, differences between *C. elegans* and mammals still exist, including differences in microbiomes. Therefore, additional studies, including chronic exposure to esculetin, are needed in order to determine potential metabolic changes, adaptations, and/or adverse effects associated with esculetin. However, despite these limitations, the findings regarding the mechanism of esculetin on lipid metabolism from the current study remain valuable for understanding its function.

## 5. Conclusions

This research demonstrated that esculetin reduced lipid accumulation dependent on IIS and AMPK signaling pathways in *C. elegans* without significantly affecting energy expenditure or food intake behaviors. The suggested signaling pathways for esculetin’s fat-lowering effect involved upregulated IIS downstream genes (*hsp-16.2* and *sod-3*), AMPK genes (*aak-1* and *aak-2*), a sirtuin gene (*sir-2.1*), and a lipolysis-related gene (*atgl-1*). Further functional analyses, such as of the effects of esculetin on mitochondrial fatty acid β-oxidation and lipolysis readouts, would support the significance of the current observations.

## Data Availability

The data presented in this study are available upon request from the corresponding author.

## Abbreviations

The following abbreviations are used in this manuscript:

AMPK	5′-Adenosine monophosphate-activated protein kinase
C/EBP	CCAAT-enhancer-binding protein
DMSO	Dimethyl sulfoxide
FUDR	Floxuridine
IGF-1	Insulin-like growth factor-1
IIS	Insulin/insulin-like growth factor-1 signaling
PPAR	Peroxisome proliferator-activated receptor
SREBP-1C	Sterol regulatory element binding protein-1C
